# A new in situ hybridization and immunohistochemistry with a novel antibody to detect *small T-antigen* expressions of Merkel cell polyomavirus (MCPyV)

**DOI:** 10.1186/1746-1596-9-65

**Published:** 2014-03-20

**Authors:** Michiko Matsushita, Daisuke Nonaka, Takeshi Iwasaki, Satoshi Kuwamoto, Ichiro Murakami, Masako Kato, Keiko Nagata, Yukisato Kitamura, Kazuhiko Hayashi

**Affiliations:** 1 Division of Molecular Pathology, Department of Pathology, Tottori University Faculty of Medicine, Yonago, Japan; 2 Department of Pathobiological Science and Technology, School of Health Science, Tottori University Faculty of Medicine, Yonago, Japan; 3 Department of Histopathology, The Christie NHS Foundation Trust, Manchester, UK

**Keywords:** Merkel cell carcinoma, MCPyV-small T antigen, ST-immunohistochemistry, *ST* mRNA-ISH

## Abstract

**Background:**

Approximately 80% of Merkel cell carcinomas (MCCs) harbor Merkel cell polyomavirus (MCPyV) which monoclonally integrates into the genome and has prognostic significance. The presence or absence of MCPyV is usually diagnosed using CM2B4 immunohistochemistry (IHC) for MCPyV-*large T antigen* (*LT*) protein. However, this method poses a risk of misdiagnosis.

**Methods:**

In this study, we determined MCPyV infection in MCCs using real-time PCR for MCPyV-*LT* DNA and prepared 16 cases of MCPyV-DNA-positive and -negative groups. Diagnostic sensitivity and specificity of conventional PCR for MCPyV-*small T antigen* (MCPyV-*ST*), IHC using a newly developed polyclonal antibody (ST-1) for MCPyV-*ST* protein (MCPyV-ST) (aa: 164–177), and in situ hybridization (ISH) as well as real-time PCR for MCPyV*-ST* mRNA were compared against CM2B4-IHC for sensitivity (0.94, 15/16) and specificity (0.94, 15/16).

**Results:**

The followings are the respective sensitivity and specificity results from examinations for MCPyV-*ST* gene: conventional PCR for the MCPyV-*ST* (0.94, 1.0), ST-1-IHC (0.69, 1.0), real-time PCR for *ST* mRNA (1.0, no data), *ST* mRNA ISH (0.94, 1.0). Each of the MCPyV-pseudonegative (1/16) and -pseudopositive (1/16) diagnoses evaluated using CM2B4-IHC were accurately corrected by examinations for MCPyV-*ST* or its expression as well as real-time PCR for MCPyV-*LT*. Sensitivity of CM2B4-IHC (0.94) was superior to that of ST-1-IHC (0.69) but equal to that of *ST* mRNA-ISH (0.94). Specificities of ST-1-IHC (1.0) and *ST* mRNA-ISH (1.0) were superior to that of CM2B4-IHC (0.94).

**Conclusions:**

Therefore, combined application of *ST* mRNA-ISH and ST-IHC as well as CM2B4-IHC is recommended and will contribute to the diagnostic accuracy for MCPyV infection in MCCs.

**Virtual slides:**

The virtual slide(s) for this article can be found here: http://www.diagnosticpathology.diagnomx.eu/vs/9966295741144834

## Background

Merkel cell carcinoma (MCC) is a rare and aggressive neuroendocrine skin cancer and Merkel cell polyomavirus (MCPyV) is monoclonally integrated into the genome of approximately 80% of MCCs [1]. The MCPyV genome contains *smallT antigen* (*ST*) and *largeT antigen* (*LT*) that encode nonstructural proteins and are responsible for viral replication and *viral proteins* (*VPs*) that constitute viral particles [2]. Although the exact pathogenesis has not yet been elucidated in MCCs, it is considered that pathogenesis of MCPyV-positive and -negative MCCs is different [2-5]. Moreover, MCPyV-positive MCCs are reported to have a better prognosis than MCPyV-negative MCCs [6-10], although these findings are some controversial [11]. Therefore, accurate diagnosis of the presence of MCPyV in MCCs is clinically important. Immunohistochemistry (IHC) using a monoclonal antibody CM2B4 that detects MCPyV-*LT* protein (MCPyV-LT) is currently the most common and prevailed method for diagnosis of MCPyV infection in MCCs, although real-time PCR is the most reliable method for confirming MCPyV-DNA and MCPyV infection in MCCs. The only commercially available antibody used for MCPyV infection diagnosis is CM2B4 antibody. IHC with CM2B4 antibody displays high sensitivity and good specificity for MCPyV detection and is usually sufficient for practical diagnosis, but it is not ideal for determining the presence or absence of MCPyV, based on reported MCC cases with pseudonegative and pseudopositive staining [3,12-14]. The *ST* gene harbors fewer mutations than the *LT* gene in MCPyV from MCCs [15], and the MCPyV-*ST* protein (MCPyV-ST) was detected in human MCC tumors more commonly than was MCPyV LT [16].

In this study, we aimed to raise the diagnostic accuracy in determining MCPyV infection in MCCs and developed a new polyclonal antibody (ST-1) for detecting MCPyV-ST (aa: 164–177) and established a new in situ hybridization (ISH) as well as real-time PCR for MCPyV-*ST* mRNA expression. The sensitivity and specificity of the newly developed methods to detect MCPyV-*ST* expressions were compared with those of CM2B4-IHC.

## Materials and methods

### MCC samples

We used 32 formalin fixed paraffin embedded (FFPE) MCC samples from 13 Japanese (MCPyV-positive: 10, MCPyV-negative: 3, from 1998 to 2008) and 19 Caucasians from the UK (MCPyV-positive: 6, MCPyV-negative: 13, from 1994 to 2007).

### Detection of MCPyV-DNA and quantification of MCPyV-*ST* mRNA expression

Real-time PCR was performed as previously described to detect and quantify MCPyV-*LT* DNA [13,17]. In addition, conventional PCR was performed using ST primer sets to detect MCPyV-*ST* DNA [14]. To quantify expression of MCPyV-*ST* mRNA, we used the Universal Probe Library Human TBP Gene Assay (Roche, Switzerland) as an internal control. After converting RNAs to cDNAs, cDNA fragments from MCPyV-*ST* mRNA and control *TBP* gene were amplified by real-time PCR using the following primer sets and probe: qST forward primer; 5′-AGTGTTTTTGCTATCAGTGCTTTATTCT-3′, qST reverse primer; 5′-CCACCAGTCAAAACTTTCCCA-3′ and fluorogenic ST probe; 5′-FAM-TGGTTTGGATTTCCTC-MGB-3′.

### IHC for MCPyV-LT detection

IHC with CM2B4 antibody (Santa Cruz Biotechnology, Inc. Dallas, TX, USA) was performed using a polymer-based method to detect MCPyV-LT [13,14].

### ST antibody (ST-1) manufacturing and IHC for MCPyV-ST detection

We established a Japanese MCPyV consensus sequence (DDBJ, Accession number: AB811689). Based on this MCPyV consensus sequence, we synthesized 164–177 amino acids and manufactured a rabbit polyclonal affinity purified antibody against MCPyV-ST (ST-1).

Staining protocol for ST-1 is the same as the one used for LT antibody (CM2B4) except for the primary and secondary antibodies. We used our primary antibody (ST-1, dilution 1/5000) and peroxidase-conjugated goat anti–rabbit IgG as a secondary antibody.

### *ST* probe and protocol for ISH

Probe against MCPyV-*ST* (nt 196–756) was produced using the CUGA ® 7 in vitro Transcription Kit (NIPPON GENE, Japan). Instead of 100 mM CTP, 100 mM UTP and 100 mM ATP provided in the kit, we used the DIG RNA Labeling Mix (Roche, Switzerland). We followed Kit manual and the *ST* probe was electrophoresed and verified as one band.

The IsHyb In Situ Hybridization (ISH) kit (BioChain, USA) and TSA PLUS DNP (HRP) SYSTEM (Perkin Elmer, USA) were used for ISH and we followed the user manuals. The protocol is described briefly as follows: After deparaffinization and rehydration, endogenous peroxidase activity was blocked using 3% hydrogen peroxide in methanol for 5 minutes (min). The slides were fixed with 4% paraformaldehyde in diethylpyrocarbonate (DEPC) –PBS at room temperature (RT) for 20 min. Slides were then washed the twice with DEPC–PBS at RT for 5 min, treated with 10 μg/ml proteinase K at 37°C for 10 min, washed once in DEPC–PBS at RT for 5 min, and fixed them again with 4% paraformaldehyde in DEPC–PBS for 15 min. Following fixation, the slides were rinsed once with DEPC–water, pre-hybridized with ready-to-use pre-hybridization solution for 4 hours at 50°C, and hybridized using digoxingenin labeled probe (3 ng/μl) in a hybridization solution for overnight (8 to16 hours) at 45°C. After washing in Saline Sodium Citrate (SSC) buffer, the slides were blocked in Tris-NaCl-blocking (TNB) buffer for 30 min at RT, incubated slides with Anti-Digoxigenin- peroxidase (POD), Fab fragments (Roche, Switzerland) for 2 hours at RT, washed in Tris-NaCl-Tween (TNT) buffer 3 times for 5 min at RT, incubated in dinitrophenyl (DNP) Amplification Reagent working solution for 10 min at RT, and washed in TNT again. The slides were incubated in anti–DNP–Horseradish Peroxidase (HRP) for 30 min at RT, washed in TNT, stained with Diaminobenzidine (DAB) for colorization, and counterstained with hematoxylin.

## Results

### Detection of MCPyV-*LT* or -LT

1) MCPyV-*LT* detection and viral load quantification using real-time PCR

Real-time PCR data were summarized in Table 1. MCC cases were divided into MCPyV DNA-positive and -negative groups based on the real-time PCR data of MCPyV-*LT*. In 16 MCPyV DNA-positive MCCs, MCPyV-*LT* viral copy numbers ranged from 0.06 to178.81 copies/cell.

**Table 1:** **Summary of MCPyV- *ST * expressions in MCPyV- *LT * DNA- positive and -negative MCCs**

			**MCPyV-*LT***		**MCPyV-*ST***				
**Case no.**	**Age (years)**	**Sex**	***LT*-DNA**	**IHC (CM2B4)**	***ST*-DNA**	***ST*-mRNA**		**IHC (ST-1)**	
			Real-time PCR*		**Conventional PCR**	Real-time PCR*	**ISH**	**Nuclear**	**Cytoplasm**
MCC32	66	M	1.20	+	+	2.11	+	++	++
MCC33	66	M	1.48	+	+	0.57	+	+	++
MCC36	87	F	0.20	+	+	4.84	+	+	-
MCC37	73	F	0.87	+	+	1.97	+	-	++
MCC47	90	M	0.06	+	+	3.13	+	-	+
MCC65	NA	F	1.50	+	+	2	+	+	+
MCC68	NA	M	11.94	+	+	13.29	+	-	+
MCC70	NA	M	14.40	+	+	4.03	+	++	+
MCC72	72	F	0.99	+	+	2.62	+	-	+
MCC74	62	M	0.20	+	-	3.14	+	-	+
MCC UK9	69	M	65.62	+	+	0.96	f+	+	-
MCC UK11	61	F	3.81	**-**	+	6.97	f+	+	++
MCC UK16	63	F	127.24	+	+	8.16	+	+	++
MCC UK19	85	F	93.19	+	+	1.11	+	+	++
MCCU K21	46	F	51.82	+	+	6.6	-	++	++
MCC UK22	74	F	178.81	+	+	1.68	+	+	+
MCC50	82	M	-	-	-	ND	-	-	+
MCC63	NA	M	-	-	-	ND	-	-	++
MCC64	NA	F	-	**f+**	-	-	-	-	+
MCC UK1	81	F	-	-	-	ND	-	-	+
MCC UK2	81	F	-	-	-	ND	-	-	++
MCC UK3	85	F	-	-	-	ND	-	-	+
MCC UK4	93	F	-	-	-	ND	-	-	+
MCC UK6	82	M	-	-	-	ND	-	-	++
MCC UK8	87	F	-	-	-	ND	-	-	+
MCC UK10	94	F	-	-	-	ND	-	-	++
MCC UK12	80	M	-	-	-	ND	-	-	+
MCC UK13	61	M	-	-	-	ND	-	-	++
MCC UK14	86	F	-	-	-	ND	-	-	++
MCC UK15	83	F	-	-	-	ND	-	-	++
MCC UK17	83	F	-	-	-	ND	-	-	+
MCC UK18	94	F	-	-	-	ND	-	-	++

*Abbreviations*: *IHC* immunohistochemistry, *ISH* in situ hybridization.

NA, not available; ND, not done; *, copies per cell; f+, focally positive.

2) Immunohistochemical detection of MCPyV-LT

**Figure 1:**
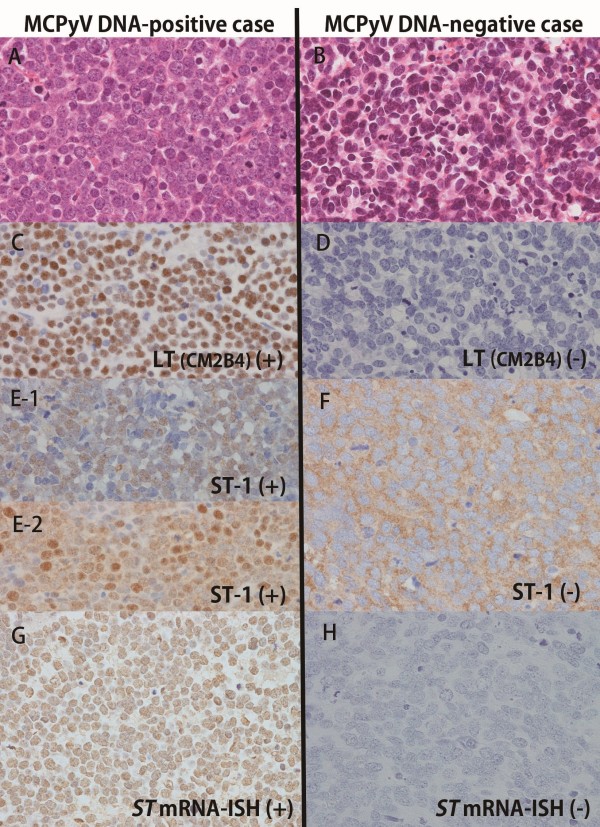
**Representative histology, immunohistochemistry and ISH of MCPyV-positive MCCs and -negative MCCs.** MCPyV-positive MCC **(A)** showed immunoreactivity for CM2B4 (MCPyV-LT) **(C)**, whereas MCPyV-negative MCC **(B)** was negative for CM2B4 **(D)**. There were two MCPyV-ST positive patterns in MCPyV-positive MCCs; 1) Nuclear immunoreactivity for ST-1 (MCPyV-ST) **(E-1)** and 2) Nuclear and cytoplasmic immunostaining for ST-1 **(E-2)**, whereas MCPyV-negative MCCs showed nuclear negativity and nonspecific cytoplasmic staining **(F)**. Nuclear positivity for ISH (MCPyV-*ST* mRNA) was observed in MCPyV-positive MCCs **(G)**, but no positive staining was detected in MCPyV-negative MCCs **(H)**. **A** and **B**, **H** and **E** stain; **A** through **H**, original magnification × 400.

**Figure 2:**
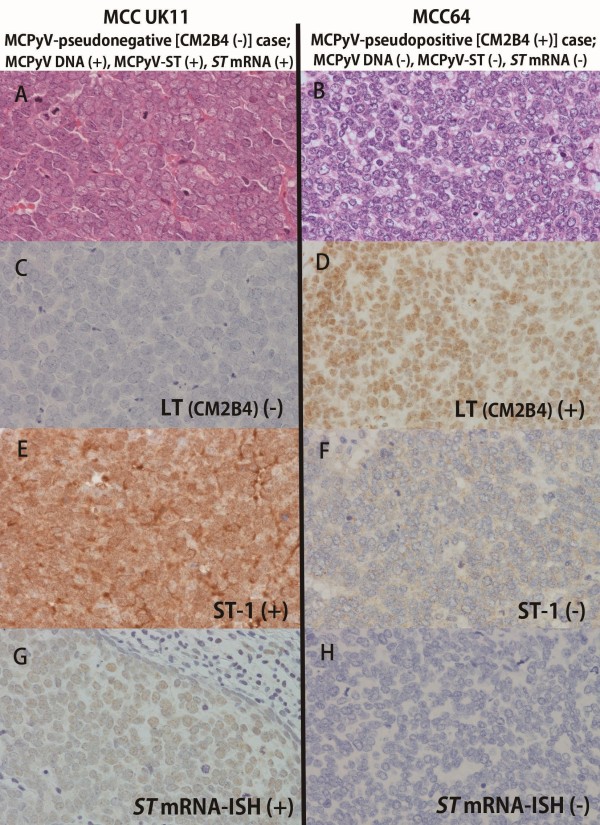
**MCPyV-pseudonegative [CM2B4 (−) and MCPyV-DNA (+)] and -pseudopositive [CM2B4 (+) and MCPyV-DNA (−)] MCCs.** MCC UK11 case **(A)** was negative for MCPyV-LT (CM2B4) **(C)** despite the presence of MCPyV-DNAs (*LT*-DNA; 3.81 copies/cell) and was positive for MCPyV-ST immunoreactivity (ST-1) **(E)** and -*ST* mRNA expression by ISH **(G)**. CM2B4-positive MCC64 **(B)** had no MCPyV-DNAs and showed no expression of ST-1 **(F)** and *ST* mRNA **(H)**. **A** and **B**, **H** and **E** stain; **A** through **H**, original magnification × 400.

The IHC results using CM2B4 were shown in Table 1 and representative cases were illustrated in Figure 1. MCPyV DNA-positive MCCs were immunoreactive for CM2B4 antibody except for one case (MCC UK11, left side of Figure 2, Figure 2C for CM2B4). MCPyV DNA of MCC UK11 was amplified using conventional PCR (*ST*-DNA) and using real-time PCR (*LT*-DNA; 3.81 copies/cell). MCPyV DNA-negative MCCs were negative for CM2B4 but one case (MCC64, right side of Figure 2) was focally CM2B4-positive (Figure 2D). As shown in Figure 2, these two cases of MCC UK11 and MCC64 examined by CM2B4-IHC were MCPyV-pseudonegative and -pseudopositive MCCs, respectively (Figure 2).

### Detection of MCPyV-*ST, −*ST or *-ST* mRNA

1) MCPyV-*ST* detection by conventional PCR

As shown in Table 1, MCPyV-*ST* DNA wasamplified using conventional PCR in MCPyV *LT*-DNA-positive MCCs except one case (MCC74) (15/16, 94%) but not in the MCPyV *LT*-negative group (0/16).

2) Immunohistochemical detection of MCPyV-ST

The IHC results using ST-1 were shown in Table 1 and representative cases were illustrated in Figure 1E. Nuclear ST (ST-1 antibody)-immunoreactivity was observed in 11 of 16 MCPyV DNA-positive MCCs (69%) but not in any of 16 MCPyV DNA-negative MCCs (0/16, 0%). Cytoplasmic ST-1 immunostaining was observed in all MCPyV DNA-positive and -negative MCCs, except two MCPyV DNA-positive cases (MCC36 and MCC UK9), which showed only nuclear immunoreactions (Figure 1E-1). Nine of 11 nuclear ST-1 positive cases in MCPyV DNA-positive MCCs also showed some extent of cytoplasmic ST-1 reaction (Figure 1E-2). These findings suggest that nuclear ST-1 immunostaining is significant and cytoplasmic ST-1 reaction is nonsignificant. The respective sensitivity and specificity of ST-1 nuclear and cytoplasmic reactions were as follows: Evaluation for nuclear reaction: sensitivity, 0.69 (11/16 DNA-positive cases); specificity, 1.0 (0/16 DNA-negative cases) and evaluation for cytoplasmic reaction: sensitivity, 0.88 (14/16 DNA-positive cases); specificity, 0 (16/16 DNA-negative cases).

3) Quantification of *ST* mRNA using real-time PCR and detection of *ST* mRNA expression using ISH

*ST* mRNA was quantified using real-time PCR only in all MCPyV DNA-positive cases and ranged from 0.57 to 13.29 copies /cell, but was undetectable in the MCPyV DNA-negative cases (Table 1).

ISH data for *ST* mRNA expression were shown in Table 1. A representative *ST* mRNA-positive case was indicated in Figure 1G. A nuclear *ST* mRNA-positive signal was observed in 15 of 16 MCPyV DNA-positive cases (sensitivity: 0.94) and in no MCPyV DNA-negative cases (0/16; specificity: 1.0, Figure 1H).

In MCPyV DNA-positive MCC cases, the frequency of *ST* mRNA expression by ISH (15/16, 94%) was higher than that of ST-1 nuclear expression (11/16, 69%). *ST* mRNA expression data by ISH did not necessarily correspond to the quantity of *ST* mRNA using real-time PCR or ST-1 nuclear immunoreaction data by IHC.

Detailed comparisons of sensitivity and specificity in all kinds of examinations for detecting MCPyV infection in MCCs were summarized in Table 2.

**Table 2:** Comparison of sensitivity and specificity among real-time PCR, conventional PCR, IHC and ISH for detecting MCPyV-infection

**Target gene**	**Kinds of examinations**	**Sensitivity**	**Specificity**
MCPyV-*LT*	Real-time PCR	1.0	1.0
	IHC (CM2B4)	0.94	0.94
MCPyV-*ST*	Conventional PCR for *ST* DNA	0.94	1.0
	IHC (ST-1)		
	Nuclear reaction	0.69	1.0
	Cytoplasmic reaction	0.88	0
	Real-time PCR for *ST* mRNA	1.0	ND
	ISH for *ST* mRNA	0.94	1.0

### Correction of MCPyV-pseudonegative (CM2B4-negative and *LT* DNA-positive) and MCPyV-pseudopositive (CM2B4-positive and *LT* DNA-negative) cases by *ST* mRNA-ISH and ST-IHC

Figure 2 illustrates an MCPyV-pseudonegative case (MCC UK11, left side) and MCPyV-pseudopositive case (MCC64, right side), which were reconfirmed as MCPyV-positive and -negative, respectively, by *ST* mRNA-ISH and ST-IHC. Sensitivity of CM2B4-IHC (0.94, 15/16) was superior to that of ST-IHC (0.69, 11/16) but equal to the sensitivity of *ST* mRNA-ISH (0.94, 15/16). Therefore, combined application of *ST* mRNA-ISH and ST-IHC as well as CM2B4-IHC is recommended and will contribute to the better and precise diagnosis of MCPyV infection in MCCs.

## Discussion

To detect MCPyV-LT, IHC along with a monoclonal antibody, CM2B4, is commonly used by several pathologists and researchers to determine infection in MCCs [14,18]. However, Kuwamoto et al. [13] pointed imperfect in sensitivity and specificity of CM2B4. Some researchers also reported CM2B4-pseudonegative or -pseudopositive MCC cases that were immunonegative for CM2B4 despite the detection of MCPyV-DNA using conventional PCR or real-time PCR [3,12,14] or immunopositive for CM2B4 despite no detection of MCPyV-DNA using conventional PCR and real-time PCR [13]. The CM2B4 antibody is extremely useful but not ideal in both sensitivity and specificity to determine accurate MCPyV infection in MCCs, because CM2B4 IHC data were not necessarily compatible with those of real-time PCR, which is the most reliable method. Recently, Leitz et al. [19] also emphasized that combined use of ST-specific antibody or a more sensitive LT-specific antibody (Ab3) and PCR using multiple primer pairs need to be applied to increase sensitivity, because CM2B4 IHC does not detect all MCPyV-positive MCC cases.

In this study, we prepared each 16 cases of MCPyV-DNA-positive and -negative MCCs which were determined by real-time PCR for MCPyV-*LT.* The sensitivity and specificity in all methods used in this study to detect MCPyV-*ST* DNA, −*ST* mRNA or -ST were summarized in Table 2, comparing with those of the popularized CM2B4 IHC. MCPyV-*ST* DNA was detected in 15 of the total 32 MCC cases (15/16 MCPyV-*LT* DNA-positive MCCs) using conventional PCR with the ST primers (sensitivity, 0.94; specificity, 1). Differences in the sensitivity of real-time or conventional PCR with the different primers may be caused by some mutations in the target region, making amplification of DNA fragments difficult. ST-1 antibody, which was newly developed to detect MCPyV-ST (aa: 164–177), was immunoreactive for the nuclei and/or cytoplasm of MCC tumor cells. According to the sensitivity and specificity data of ST-1 IHC (Nuclear reaction: sensitivity, 0.69, specificity, 1.0; cytoplasmic reaction: sensitivity, 0.875, specificity, 0), evaluation of nuclear ST (ST-1 antibody)-immunoreactivity was significant and useful for diagnosis of MCPyV-infected MCCs but cytoplasmic ST immunoreactivity was not significant. Shuda et al. also reported [16] that expression of MCPyV-ST was detected in both nuclei and cytoplasm by IHC. MCPyV*-LT* region has a nuclear localization signal (NLS) that is located between the retinoblastoma protein (RB)-binding and DNA helicase domains [20]. Therefore, nuclear LT (CM2B4 antibody)-immunoreactivity is evaluated as significant. On the other hand, NLS of MCPyV*-ST* region has not been revealed until now. Therefore, it may be possible that cytoplasmic staining of MCPyV-positive MCCs may be a partly true immunoreaction, although cytoplasmic immunoreactions in both MCPyV-positive MCCs and -negative MCCs suggest that they are not significant. In each case of MCPyV-pseudonegative (MCC UK11) or -pseudopositive (MCC64) MCC determined by CM2B4 IHC, nuclear ST-1 imunoreactivity corresponded to the data of real-time PCR for *LT*-DNA, conventional PCR for *ST*-DNA, and real-time PCR or ISH for *ST* mRNA. This was useful for correcting the misdiagnosis for MCPyV infection evaluated by CM2B4 IHC. As another method of detecting MCPyV-*ST* mRNA expression, we developed a new ISH approach using the *ST* probe recognizing MCPyV-*ST* (nt: 196–756). ISH revealed nuclear positive staining demonstrated in all MCPyV-positive MCCs except in one case (sensitivity, 0.94; specificity, 1.0). This ISH is also a good method and the first application to detect the MCPyV infection in FFPE samples of MCCs. The reason why one *ST* mRNA could not be detected by ISH was because of the old issue of FFPE. Real-time PCR for quantifying *ST* mRNA expression demonstrated the best sensitivity (1.0) and is comparable with the sensitivity (1.0) of real-time PCR for *LT* DNA. However, quantities of MCPyV-*ST* mRNA expression using real-time PCR in MCPyV-infected MCCs did not necessarily correlate with the expression levels of *ST* mRNA-ISH or ST-1 IHC. With regard to the studies of MCCs, MCPyV*-LT* is the main analytic region in the studies of MCPyV-infected MCCs because MCPyV-DNA with truncated mutations in the *LT* region is integrated into the MCC host genome [21]. The interaction of MCPyV*-*LT and RB [22] is essential for sustaining tumor growth. A recent study [16] indicated that MCPyV*-ST* expression was required for the Merkel cell tumor growth in vitro and was observed to act downstream in the mammalian target of rapamycin (mTOR) signaling pathway to preserve eukaryotic translation initiation factor 4E-binding protein 1 (4E-BP1) hyperphosphorylation, resulting in dysregulated cap-dependent translation and that 4E-BP1 inhibition is required for MCPyV transformation. The function of MCPyV*-ST* has not been fully elucidated and in order to analyze this in the future, ST-1 IHC and *ST* mRNA-ISH will be useful methods.

## Conclusion

In conclusion, we emphasize again that in order to precisely determine MCPyV infection of MCCs, combined application of ST-1 IHC and *ST* mRNA-ISH as well as CM2B4 IHC is recommended and will contribute to a better and accurate diagnosis for MCPyV infection in MCCs.

## Competing interests

The authors declare that they have no competing interests.

## Authors’ contributions

MM, TI and KH designed the study and wrote the manuscript. DN collected the samples, patients’ information and follow-up data in United Kingdom. SK, IM, MK, KN and YK collected Japanese samples and joined the study. All authors have read and approved the final manuscript.
